# Association of Interpregnancy Interval With Adverse Birth Outcomes

**DOI:** 10.1001/jamanetworkopen.2022.16658

**Published:** 2022-06-13

**Authors:** Ting Xu, Huazhang Miao, Yuliang Chen, Limei Luo, Pi Guo, Yingxian Zhu

**Affiliations:** 1 Department of Preventive Medicine, Shantou University Medical College, Shantou, China; 2 Department of Healthcare, Guangdong Women and Children Hospital, Guangzhou, China; 3 Department of Medical Quality Management, Nanfang Hospital, Guangzhou, China; 4 Department of Radiation Oncology, Cancer Hospital of Shantou University Medical College, Shantou, China; 5 Guangdong Provincial Key Laboratory of Infectious Diseases and Molecular Immunopathology, Shantou, China

## Abstract

**Importance:**

Many studies have reported an association of interpregnancy interval (IPI) between 2 consecutive births with adverse birth outcomes in low- and middle-income countries. However, most of these studies ignore the implications of some unmeasured confounders.

**Objective:**

To explore the association of IPI with adverse perinatal outcomes.

**Design, Setting, and Participants:**

This large-scale cohort study used the Guangdong Provincial Women and Children Health Information System in Guangdong Province, China, to obtain birth data recorded between January 1, 2014, and December 31, 2020. Matched-sibling design was used. The final cohort included first-born and second-born sibling pairs delivered by mothers who were permanent residents of Guangdong Province.

**Exposures:**

The exposure variable was IPI, which was categorized as follows: less than 6, 6 to 11, 12 to 17, 18 to 23, 24 to 29, 30 to 35, and 36 or more months.

**Main Outcomes and Measures:**

The outcome variables were adverse birth outcomes: preterm birth (PTB, gestational age <37 weeks), low birth weight (LBW, <2500 g), and small for gestational age (SGA). Adjusted odds ratio (OR) and interaction odds ratio (IOR) associated with IPI were calculated.

**Results:**

The study consisted of 725 392 sibling pairs of multiparous mothers. Among these mothers, 718 111 (99.0%) were aged 20 to 34 years, and 715 583 (98.7%) were of Han Chinese ethnicity. Unmatched analysis showed that a short IPI of less than 6 months was associated with higher risks of PTB (adjusted OR, 1.96; 95% CI, 1.87-2.06), LBW (adjusted OR, 1.88; 95% CI, 1.79-1.98), and SGA (adjusted OR, 1.34; 95% CI, 1.30-1.38) compared with an IPI of 18 to 23 months. These associations were attenuated in the matched-sibling analysis. An association of short IPI (<6 months) with PTB (adjusted IOR, 1.40; 95% CI, 1.30-1.51), LBW (adjusted IOR, 1.30; 95% CI, 1.21-1.40), and SGA (adjusted IOR, 1.16; 95% CI, 1.11-1.22) remained in the matched analysis. For IPI of 36 months or more, the odds of PTB (adjusted OR, 1.08; 95% CI, 1.03-1.14) and LBW (adjusted OR, 1.13; 95% CI, 1.07-1.19) in the unmatched analysis were also greater than the reference interval (18-23 months), but not for SGA (adjusted OR, 0.96; 95% CI, 0.93-0.99). Associations between a long IPI (≥36 months) and PTB (adjusted IOR, 1.10; 95% CI, 1.02-1.19) and LBW (adjusted IOR, 1.16; 95% CI, 1.07-1.26) remained through the sibling comparisons.

**Conclusions and Relevance:**

Results of this study indicated that mothers with a short (<6 months) or long (≥36 months) IPI had greater odds of adverse birth outcomes. The findings may inform family planning policies and guide individuals and families who are planning for another pregnancy in China.

## Key Points

**Question:**

How long is the optimal interpregnancy interval (IPI), and what is the association between IPI and subsequent adverse birth outcomes?

**Findings:**

In this cohort study of 725 392 sibling pairs in Guangdong Province, China, short or long IPI was associated with increased odds of adverse birth outcomes.

**Meaning:**

Findings of this study may inform family planning policies and provide prepregnancy advice to those who are planning for another pregnancy in China.

## Teaser

This cohort study assesses the association of interpregnancy interval with low birth weight, small for gestational age, and preterm birth outcomes among sibling pairs in China.

## Introduction

Adverse birth outcomes, such as low birth weight (LBW), small for gestational age (SGA), and preterm birth (PTB), have raised public concerns worldwide.^1^ Low birth weight, SGA, and PTB are associated with neonatal mortality,^2,3,4^ and adverse birth outcomes have detrimental consequences for health later in life.^5^ Therefore, it is imperative to investigate the potential risk factors for adverse perinatal outcomes.

Several studies have proposed that interpregnancy interval (IPI) is a potential modifiable risk factor for adverse birth outcomes.^6^ Epidemiological studies have demonstrated that short IPI is associated with increased risks of adverse perinatal outcomes.^7,8,9^ The World Health Organization has stressed the importance of exploring the association between adverse perinatal outcomes and IPI in future research.^10^

A number of studies investigated whether IPI is associated with PTB, LBW, and SGA.^11,12,13^ However, their results were inconsistent. Variation in these findings may be attributed to differences in medical care, industrial development, and maternal characteristics. Thus, research in different locations can be beneficial for a comprehensive understanding of how IPI affects adverse birth outcomes. In addition, most studies on IPI and adverse birth outcomes are traditional retrospective cohort studies that did not adjust for certain unmeasured confounders.^14,15^ Not adjusting for confounders may raise the possibility of underestimating or overestimating the association between IPI and birth outcomes of interest. According to previous studies, IPI is associated with many potential confounders, such as maternal age, ethnicity, and socioeconomic status.^16^ For example, short IPIs are more common among young mothers.^17^ It is unclear to what extent this association is attributed to some unmeasured confounding factors. Several studies involving populations in high-income countries used a sibling-matched design to account for some unmeasured confounders, such as genetic factors and lifestyles.^18,19,20,21^ However, only a few studies explored the association of IPI with adverse birth outcomes among the Chinese population.^22,23^ Nevertheless, these studies did not account for some unmeasured confounders.

In 2015, China’s 1-child policy was replaced with a universal 2-child policy. In 2021, a 3-child policy was announced in the country in response to the decline in total fertility rates.^24^ In the context of changes in the birth policy, more families may consider having another child. Therefore, it is vital to formulate guidelines for appropriate IPI that are based on evidence obtained from large-scale cohort studies involving Chinese populations. With large-scale population data collected from Guangdong Province in China, we conducted this cohort study to explore the association of IPI with adverse perinatal outcomes using a matched-sibling design.

## Methods

This study was approved by the Medical Ethics Committee at the Guangdong Women and Children Hospital. The birth surveillance data used in this study were extracted without personally identifiable information; therefore, the study was considered to be exempt from informed consent by the Medical Ethics Committee. We followed the Strengthening the Reporting of Observational Studies in Epidemiology (STROBE) reporting guideline.

### Study Population

We obtained data from the Guangdong Provincial Women and Children Health Information System, which collects information on newborns and mothers from all medical institutions in this region. After birth, obstetric medical staff measure the neonate’s weight with an electronic scale (weighing accuracy within 1 g). Gestational age (weeks) is then ascertained by obstetricians using the date of the mother’s last menstrual period and ultrasonographic examination. The information is checked by quality control physicians or nurses from the regional medical facility before being submitted to the Health Information System. Professional medical workers verify the accuracy of the information. The birth registration database in the Health Information System includes the neonate’s birth date, birth weight, and sex; maternal age and ethnicity; mode of delivery; and gestational age.

**Figure 1.**
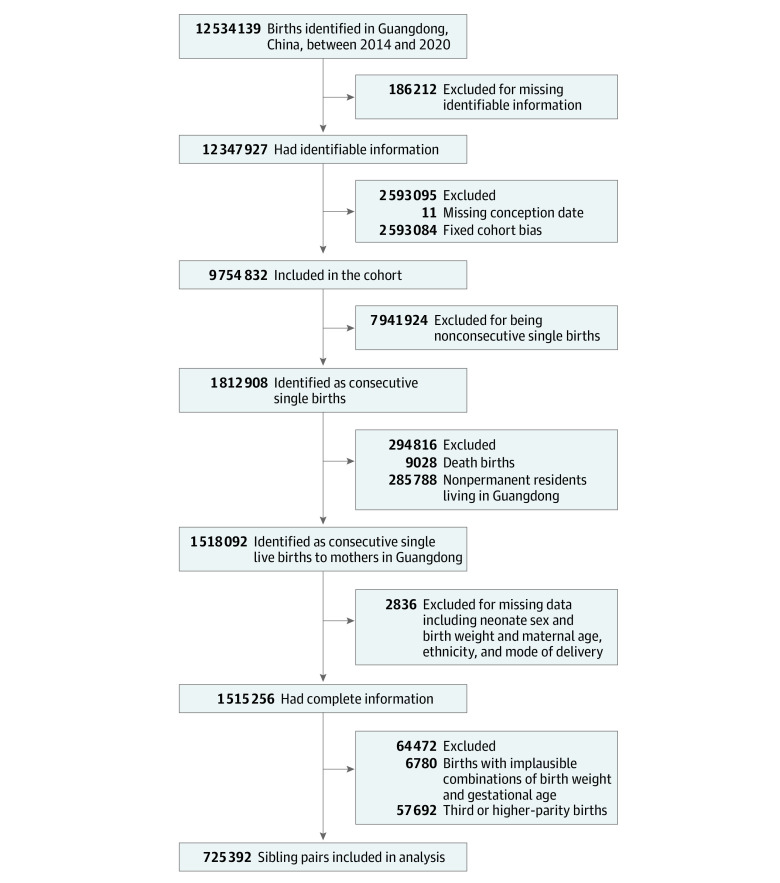
Flowchart of Selection of Births in Guangdong Province From 2014 to 2020

There were 12 534 139 births recorded in the database from January 1, 2014, to December 31, 2020 (Figure 1). Of these, 12 347 927 births had complete identifiable information. We excluded births with missing conception dates (n = 11) and conception dates of 42 weeks before the study end date (December 31, 2020; n = 2 593 084) to avoid fixed cohort bias. Fixed cohort bias arises in retrospective cohorts with a specific time window, thereby missing shorter pregnancies at the beginning of the study and longer pregnancies at the end of the study.^25^ Furthermore, we excluded nonconsecutive single births (n = 7 941 924). We restricted births to mothers who permanently resided in Guangdong Province (n = 1 518 092). After exclusion of births with missing information, including neonate’s sex and birth weight, maternal age and ethnicity (as recorded in the database and including Han Chinese and 55 ethnic minority groups), and mode of delivery (n = 2836), 1 515 256 births with complete information remained. Births with implausible weight (<500 g or >5000 g) or gestational age (<20 weeks or >42 weeks [n = 6780]) and births at third or higher parity (n = 57 692) were also excluded. The final cohort included 725 392 first-born and second-born sibling pairs.

### Exposure and Outcome Definitions

Interpregnancy interval, defined as the period between the delivery date for one child and the conception date for the next child, was the exposure variable. We calculated IPI by subtracting the gestational age of the second birth from the time elapsed between the first and second deliveries. Following previous research,^17,22^ we classified IPI into 7 categories (<6, 6-11, 12-17, 18-23, 24-29, 30-35, ≥36 months), and the 18- to 23-month category was used as the reference group.

Adverse birth outcomes of LBW (<2500 g), PTB (gestational age <37 weeks), and SGA were used as the dependent or outcome variables. Low birth weight was classified into 2 subcategories: very LBW (<1500 g) and extremely LBW (<1000 g).^26^ Preterm birth was grouped by moderate PTB (33-36 weeks), very PTB (28-32 weeks), and extreme PTB (<28 weeks).^21^ Small for gestational age was defined as birth weight below the 10th percentile of the Chinese fetal growth gestational age and sex-specific reference.^27^ Because this reference applies only to neonates with a gestational age of 24 to 42 weeks, newborns with a gestational age less than 24 weeks were excluded from the SGA analysis.

In accordance with previous studies,^22,28^ several potential confounders were considered: maternal age at first birth, maternal ethnicity, mode of delivery at first birth, season of birth, and socioeconomic status. Socioeconomic status was derived in 2 steps. First, we collected the mean income of each city from the Statistics Bureau of Guangdong Province as the income of mothers living in the same region. Second, we grouped the income into quintiles according to the population in Guangdong Province.

### Statistical Analysis

We described the basic characteristics of mothers by IPI category (<6, 6-11, 12-17, 18-23, 24-29, 30-35, ≥36 months), and then we compared the differences between the groups using χ^2^ test. Because the outcome variables were binary data, we used a logistic regression model to estimate the odds ratios (ORs) and corresponding 95% CIs for adverse perinatal outcomes and IPI, with 18 to 23 months as the reference group. In the unmatched analysis, we fitted an unadjusted model containing only IPI and an adjusted model including maternal age, maternal ethnicity, season of birth, socioeconomic status, and mode of delivery.

We used a matched-sibling design, which was proposed in a previous study,^21^ to control for some unmeasured confounding factors. With this method, we matched the first 2 consecutive births with different birth outcomes from the same mother with a discordant sibling pair (defined as a sibling pair with only 1 sibling having the adverse outcome of interest [LBW, PTB, or SGA]). The information of first birth was not incorporated into the model in the unmatched analysis. However, in the matched analysis, the information was used to adjust some unmeasured confounders. For matched analysis, we adopted an interaction term for birth order (equal to 0 for the first birth and 1 for the second birth) multiplied by IPI in the conditional logistic model to ensure that only the association between adverse birth outcomes and IPI for the second birth was estimated. Detailed methods are provided in the eAppendix in the Supplement. In the matched analysis, we fitted an unadjusted model including only IPI and an adjusted model considering factors that could change over time, such as maternal age, mode of delivery, neonate’s sex, and season of birth. This model did not include socioeconomic status because we collected only data on mothers during their first birth.

In addition, we conducted several sensitivity analyses and subgroup analyses according to maternal age and mode of delivery. We constructed a model without adjusting for delivery mode. We also conducted supplemental analyses of subcategories of PTB and LBW.

Analyses of data were conducted with SAS, version 9.4 (SAS Institute Inc). Statistical significance was denoted by 2-tailed *P* < .05.

## Results

### Baseline Characteristics

A total of 725 392 sibling pairs from multiparous mothers were included in the study. Of the sibling pairs, 181 172 second-born siblings (25.0%) were born with an IPI of 12 to 17 months. Of the mothers, 718 111 (99.0%) were aged 20 to 34 years, 715 583 (98.7%) were of Han ethnicity, 49 485 (6.8%) had an IPI of less than 6 months, and 54 988 (7.6%) had a long IPI of 36 months or more. We observed a shorter IPI of less than 6 months for mothers aged 20 to 24 years (30 400 [61.4%]), of Han Chinese ethnicity (48 966 [99.0%]), who delivered their first child in autumn (14 800 [29.9%]), and who had a vaginal delivery (48 368 [97.7%]). Mothers with a socioeconomic status in the highest quintile (17 168 [31.2%]) tended to have a longer IPI (≥36 months). Characteristics of the mothers at their first pregnancy based on IPI categories are presented in Table 1.

**Table 1.** Maternal Characteristic According to Interpregnancy Interval in Sibling Pairs, Guangdong Province From 2014 to 2020

Characteristic	Interpregnancy interval, No. (%)								*P* value
	All (N = 725 392)	<6 mo (n = 49 485)	6-11 mo (n = 174 675)	12-17 mo (n = 181 172)	18-23 mo (n = 122 866)	24-29 mo (n = 86 842)	30-35 mo (n = 55 364)	≥36 mo (n = 54 988)	
Maternal age at first birth, y									
20-24	374 582 (51.6)	30 400 (61.4)	99 926 (57.2)	95 091 (52.5)	59 918 (48.8)	40 026 (46.1)	24 883 (44.9)	24 338 (44.3)	<.001
25-29	28 8325 (39.8)	15 984 (32.3)	62 394 (35.7)	70 906 (39.1)	51 384 (41.8)	37 884 (43.6)	24 690 (44.6)	25 083 (45.6)	
30-34	55 204 (7.6)	2667 (5.4)	10 824 (6.2)	13 444 (7.4)	10 226 (8.3)	7900 (9.1)	5128 (9.3)	5015 (9.1)	
≥35	7281 (1.0)	434 (0.9)	1531 (0.9)	1731 (1.0)	1338 (1.1)	1032 (1.2)	663 (1.2)	552 (1.0)	
Maternal ethnicity^a^									
Han	715 583 (98.7)	48 966 (99.0)	172 458 (98.7)	178 801 (98.7)	121 171 (98.6)	85 578 (98.5)	54 504 (98.5)	54 105 (98.4)	<.001
Other^b^	9809 (1.4)	519 (1.1)	2217 (1.3)	2371 (1.3)	1695 (1.4)	1264 (1.5)	860 (1.6)	883 (1.6)	
Mode of delivery at first birth									
Cesarean delivery	120 809 (16.7)	1117 (2.3)	10 440 (6.0)	23 651 (13.1)	26 685 (21.7)	24 260 (27.9)	16 870 (30.5)	17 786 (32.4)	<.001
Vaginal delivery	604 583 (83.4)	48 368 (97.7)	164 235 (94.0)	157 521 (87.0)	96 181 (78.3)	62 582 (72.1)	38 494 (69.5)	37 202 (67.7)	
Socioeconomic status, quintile									
1 (Lowest)	135 798 (18.7)	9760 (19.7)	35 932 (20.6)	35 208 (19.4)	22 354 (18.2)	15 124 (17.4)	9039 (16.3)	8381 (15.2)	<.001
2	146 563 (20.2)	13 101 (26.5)	40 168 (23.0)	37 401 (20.6)	23 113 (18.8)	15 258 (17.6)	9033 (16.3)	8489 (15.4)	
3	151 635 (20.9)	10 571 (21.4)	37 311 (21.4)	39 633 (21.9)	25 842 (21.0)	17 589 (20.3)	10 765 (19.4)	9924 (18.1)	
4	145 584 (20.1)	9714 (19.6)	33 878 (19.4)	36 494 (20.1)	25 316 (20.6)	17 725 (20.4)	11 431 (20.7)	11 026 (20.1)	
5 (Highest)	145 812 (20.1)	6339 (12.8)	27 386 (15.7)	32 436 (17.9)	26 241 (21.4)	21 146 (24.4)	15 096 (27.3)	17 168 (31.2)	
Season of first birth									
Spring	177 222 (24.4)	10 051 (20.3)	44 490 (25.5)	40 364 (22.3)	31 744 (25.8)	20 380 (23.5)	15 007 (27.1)	15 186 (27.6)	<.001
Summer	179 468 (24.7)	11 717 (23.7)	46 237 (26.5)	41 135 (22.7)	33 472 (27.2)	19 169 (22.1)	15 681 (28.3)	12 057 (21.9)	
Autumn	182 389 (25.1)	14 800 (29.9)	41 776 (23.9)	50 608 (27.9)	28 952 (23.6)	22 982 (26.5)	12 177 (22.0)	11 094 (20.2)	
Winter	186 313 (25.7)	12 917 (26.1)	42 172 (24.1)	49 065 (27.1)	28 698 (23.4)	24 311 (28.0)	12 499 (22.6)	16 651 (30.3)	

^a^ Ethnicity was recorded in the data source.

^b^ Including 55 ethnic minority groups in China.

### Unmatched Analysis 

Odds of adverse birth outcomes by IPI in the unmatched analysis are presented in Table 2. Compared with the second-born sibling after an IPI of 18 to 23 months, those born after a short IPI had greater odds of PTB (<6 months: adjusted OR, 1.96 [95% CI, 1.87-2.06]; 6-11 months: adjusted OR, 1.32 [95% CI, 1.27-1.37]). Similar results were found for moderate PTB, very PTB, and extreme PTB. Short IPI of less than 6 months was associated with very PTB (adjusted OR, 3.33; 95% CI, 2.93-3.79) (eTable 1 in the Supplement). For IPI of 36 months or more, the odds of PTB were 1.08 (95% CI, 1.03-1.14).

**Table 2.** Risks of Adverse Birth Outcomes in Unmatched Cohort Analysis by Interpregnancy Interval, Guangdong Province From 2014 to 2020

Birth outcome and interpregnancy interval, mo	No. of sibling pairs	Second-born siblings with outcome, No. (%)	OR (95% CI)	
			Crude^a^	Adjusted^b^
Preterm birth				
<6	49 485	3135 (6.3)	1.71 (1.63-1.79)	1.96 (1.87-2.06)
6-11	174 675	7801 (4.5)	1.18 (1.14-1.23)	1.32 (1.27-1.37)
12-17	181 172	7116 (3.9)	1.03 (1.00-1.07)	1.1 (1.06-1.14)
18-23	122 866	4678 (3.8)	1 [Reference]	1 [Reference]
24-29	86 842	3421 (3.9)	1.04 (0.99-1.08)	0.99 (0.94-1.03)
30-35	55 364	2219 (4.0)	1.06 (1.00-1.11)	0.98 (0.93-1.03)
≥36	54 988	2519 (4.6)	1.21 (1.15-1.28)	1.08 (1.03-1.14)
Low birth weight				
<6	49 485	2913 (5.9)	1.77 (1.69-1.86)	1.88 (1.79-1.98)
6-11	174 675	7346 (4.2)	1.24 (1.20-1.29)	1.32 (1.27-1.37)
12-17	181 172	6493 (3.6)	1.05 (1.01-1.10)	1.09 (1.05-1.13)
18-23	122 866	4190 (3.4)	1 [Reference]	1 [Reference]
24-29	86 842	2881 (3.3)	0.97 (0.93-1.02)	0.95 (0.90-1.00)
30-35	55 364	1894 (3.4)	1.00 (0.95-1.06)	0.97 (0.92-1.02)
≥36	54 988	2197 (4.0)	1.18 (1.12-1.24)	1.13 (1.07-1.19)
Small for gestational age				
<6	49 444	7472 (15.1)	1.49 (1.44-1.53)	1.34 (1.30-1.38)
6-11	174 611	23 522 (13.5)	1.30 (1.27-1.33)	1.21 (1.18-1.23)
12-17	181 121	21 338 (11.8)	1.11 (1.09-1.14)	1.07 (1.05-1.10)
18-23	122 844	13 145 (10.7)	1 [Reference]	1 [Reference]
24-29	86 829	8770 (10.1)	0.94 (0.91-0.97)	0.97 (0.94-1.00)
30-35	55 350	5390 (9.7)	0.90 (0.87-0.93)	0.96 (0.93-0.99)
≥36	54 983	5175 (9.4)	0.87 (0.84-0.90)	0.96 (0.93-0.99)

Abbreviation: OR, odds ratio.

^a^ ORs and corresponding 95% CIs were based on logistic regression.

^b^ Adjusted for maternal age, maternal ethnicity, socioeconomic status, mode of delivery, neonate’s sex, and season of birth.

Significant differences were observed between short and long IPIs and LBW (<6 months: adjusted OR, 1.88 [95% CI, 1.79-1.98]; ≥36 months: adjusted OR, 1.13 [95% CI, 1.07-1.19]). Similar results were found between subcategories of LBW and IPI. We observed an association between extremely LBW and IPI after 36 months (adjusted OR, 3.06; 95% CI, 1.45-6.45) (eTable 1 in the Supplement). When we examined the associations between IPI and SGA, we found that mothers with a short IPI had greater odds of SGA (<6 months: adjusted OR, 1.34 [95% CI, 1.30-1.38]; 6-11 months: adjusted OR, 1.21 [95% CI, 1.18-1.23]; 12-17 months: adjusted OR, 1.07 [95% CI, 1.05-1.10]). Odds of SGA were not greater after an IPI of 36 months or more (adjusted OR, 0.96; 95% CI, 0.93-0.99). No evidence was found of associations between SGA and IPI longer than 24 months.

### Matched-Sibling Analysis 

**Figure 2.**
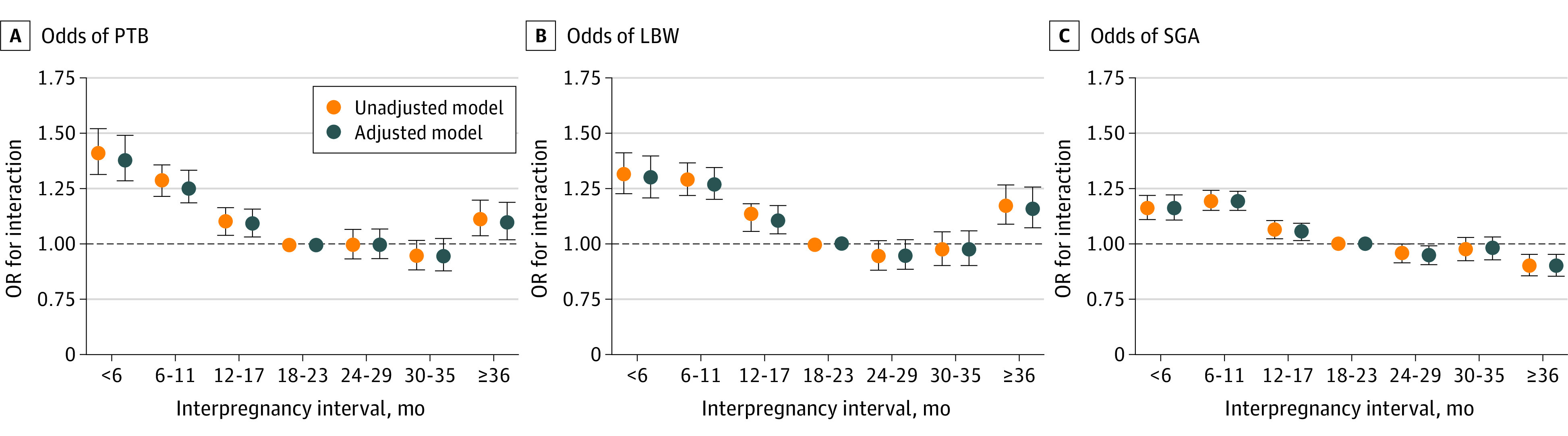
Risk of Adverse Birth Outcomes in Matched-Sibling Analysis by Interpregnancy Interval in Guangdong Province From 2014 to 2020 LBW indicates low birth weight; OR, odds ratio; PTB, preterm birth; and SGA, small for gestational age.

Odds of adverse birth outcomes by IPI in the matched-sibling analysis are illustrated in Table 3 and Figure 2. Increased odds of PTB were found for short IPIs (<6 months: adjusted interaction odds ratio [IOR], 1.40 [95% CI, 1.30-1.51]; 6-11 months: adjusted IOR, 1.27 [95% CI, 1.20-1.34]). As we investigated the association between subcategories of PTB and IPI (eTable 2 in the Supplement), we found that the result for moderate PTB was similar to all PTBs. However, we observed no association between extreme PTB and a short IPI of less than 18 months. Mothers who experienced short or long IPI had greater odds of LBW (<6 months: adjusted IOR, 1.30 [95% CI, 1.21-1.40]; ≥36 months: adjusted IOR, 1.16 [95% CI, 1.07-1.26]). Associations between long IPI (≥36 months) and PTB (adjusted IOR, 1.10; 95% CI, 1.02-1.19) were observed in sibling analysis.

**Table 3.** Risks of Adverse Birth Outcomes in Matched-Sibling Analysis by Interpregnancy Interval, Guangdong Province From 2014 to 2020

Birth outcome and interpregnancy interval, mo	No. of sibling pairs	No. of discordant sibling pairs in analysis^a^	IOR (95% CI)	
			Crude^b^	Adjusted^c^
Preterm birth				
<6	49 485	4450	1.43 (1.33-1.54)	1.40 (1.30-1.51)
6-11	174 675	11 872	1.30 (1.22-1.37)	1.27 (1.20-1.34)
12-17	181 172	11 677	1.11 (1.05-1.17)	1.10 (1.04-1.17)
18-23	122 866	7940	1 [Reference]	1 [Reference]
24-29	86 842	5823	1.00 (0.93-1.07)	1.00 (0.94-1.07)
30-35	55 364	3856	0.95 (0.88-1.03)	0.95 (0.87-1.02)
≥36	54 988	3992	1.12 (1.04-1.21)	1.10 (1.02-1.19)
Low birth weight				
<6	49 485	4780	1.32 (1.23-1.42)	1.30 (1.21-1.40)
6-11	174 675	12 792	1.29 (1.22-1.37)	1.27 (1.20-1.35)
12-17	181 172	12 171	1.12 (1.06-1.18)	1.11 (1.05-1.18)
18-23	122 866	8339	1 [Reference]	1 [Reference]
24-29	86 842	5955	0.95 (0.89-1.01)	0.95 (0.89-1.02)
30-35	55 364	3743	0.98 (0.90-1.06)	0.98 (0.90-1.06)
≥36	549 88	3969	1.17 (1.09-1.27)	1.16 (1.07-1.26)
Small for gestational age				
<6	49 444	11 143	1.17 (1.11-1.22)	1.16 (1.11-1.22)
6-11	174 611	35 835	1.20 (1.16-1.24)	1.19 (1.15-1.24)
12-17	181 121	34 971	1.06 (1.03-1.10)	1.05 (1.02-1.09)
18-23	122 844	22 455	1 [Reference]	1 [Reference]
24-29	86 829	15 333	0.95 (0.91-1.00)	0.95 (0.91-0.99)
30-35	55 350	9448	0.97 (0.93-1.02)	0.98 (0.93-1.03)
≥36	54 983	9427	0.90 (0.86-0.95)	0.90 (0.86-0.95)

Abbreviation: IOR, interaction odds ratio.

^a^ Sibling pairs with only 1 birth resulting in an adverse outcome under analysis.

^b^ Odds of second-born sibling being delivered with adverse outcome under analysis vs odds of first-born sibling being delivered with same adverse outcome.

^c^ Adjusted for maternal age, mode of delivery, neonate’s sex, and season of birth.

We observed similar results for very LBW but different results for extremely LBW. Association with no difference was found between extremely LBW and a short IPI of less than 18 months (eTable 2 in the Supplement). We observed that SGA was more likely to occur in neonates born after short IPIs (<6 months: adjusted IOR, 1.16 [95% CI, 1.11-1.22]; 6-11 months: adjusted IOR, 1.19 [95% CI, 1.15-1.24]).

As shown in Figure 2, a U-shaped association was observed between IPI and the risks of PTB and LBW, with the lower risks of these outcomes for IPI at 18 to 23 months and the higher risks for short and long IPIs (<6 and ≥36 months). Moreover, we observed attenuated associations of IPI with PTB, LBW, and SGA in the adjusted model vs unadjusted model.

### Sensitivity Analysis

Results of a traditional logistic analysis that was restricted to discordant sibling pairs are presented in eTable 3 in the Supplement, which can be used for comparisons. The analysis showed that IPIs of less than 6 months were associated with increased risks of PTB (adjusted OR, 1.58; 95% CI, 1.47-1.71), LBW (adjusted OR, 1.42; 95% CI, 1.32-1.53), and SGA (adjusted OR, 1.17; 95% CI, 1.12-1.23). We found that, without adjusting for delivery (eTable 4 and eTable 5 in the Supplement), the estimates in the model were not substantially changed compared with the main analysis. Subgroup analysis stratified by mode of delivery is displayed in eFigure 1 and by maternal age in eFigure 2 in the Supplement. We observed slight but not meaningful increases in the odds of adverse birth outcomes at a short IPI of less than 12 months for mothers with previous cesarean delivery compared with vaginal delivery. Odds of PTB and LBW at a short IPI of less than 6 months were increased for mothers younger than 35 years and, to a lesser extent, those older than 35 years.

## Discussion

To our knowledge, this population-based study was the largest analysis of the association between IPI and adverse perinatal birth outcomes ever conducted in China. Moreover, the matched-sibling method was used for the first time to investigate this association. We found that a short IPI of less than 6 months was associated with increased risks of adverse birth outcomes such as PTB, LBW, and SGA. Mothers with a long IPI of 36 months or more had greater odds of PTB and LBW in the second delivery. We also found that PTB and LBW showed a U-shaped association with IPI. The result of the adjusted model was similar to the unadjusted model, which is consistent with a previous study that reported maternally related factors might not be necessary to adjust for confounders when examining the implications of short interpregnancy periods for pregnancy outcomes.^29^ In accordance with previous matched-sibling studies, this study found attenuated associations of IPI with adverse perinatal outcomes in the matched analysis compared with unmatched analysis, suggesting that associations may be partially explained by several unmeasured confounders.

According to the unmatched analysis, there were increased odds of PTB, LBW, and SGA after a short IPI of less than 6 months, which was in line with findings from previous research.^22,30,31,32^ In the US, Lonhart et al^12^ found greater risks of PTB with a short IPI of less than 6 months. In Canada, Schummers et al^8^ found that a short IPI of less than 6 months was associated with increased risks of SGA. In accordance with the previous research, we came to similar conclusions. However, a few unmatched studies in China have presented inconsistent results compared with the unmatched analysis. For example, Shi et al^23^ used a generalized linear model to explore the association of IPI with adverse perinatal outcomes in Northwest China; they found that a shorter IPI of less than 6 months was associated with higher risks of SGA but not PTB and LBW. A possible explanation for such differences is that different sample sizes and adjustment factors were involved. For the unmatched analysis, the OR of the adjusted model was slightly higher than the OR of the unadjusted model, which was also observed in previous studies.^21,33^ We speculated that some unmeasured confounding factors might be contributing to the associations between IPI and adverse birth outcomes.

Moreover, results of the subgroup analysis suggested that increased odds of adverse birth outcomes after a short IPI of less than 6 months were more pronounced for mothers younger than 35 years than those aged 35 years or older; a similar result was reported in a previous study.^8^ Several studies^28,34^ investigated how IPI and adverse birth outcomes varied by previous delivery mode, but their scope was limited. The present study indicated that mothers with cesarean delivery at first birth had modest increases in the odds of adverse birth outcomes than mothers with vaginal delivery, but the odds did not vary significantly.

The mechanisms of IPI and adverse perinatal birth outcomes remain unknown. Several hypotheses, including nutritional depletion and anemia, have been proposed as explanations for short IPI and adverse birth outcomes.^35,36^ According to the nutritional depletion hypothesis, mothers are not given sufficient time to recover from malnutrition after the first pregnancy and subsequent breastfeeding.^37,38^ For example, if physiologic iron intake is not adequate during pregnancy or breastfeeding, future pregnancies are at risk for iron deficiency, leading to LBW and PTB.^39,40^ Although the mechanism explaining the association between a long IPI and adverse birth outcomes remains unclear, physiologic regression may account for this association. According to this hypothesis, previous pregnancy may have provided benefits to mothers. However, there may be a slow decline in benefits after delivery until a mother's physiologic characteristics are in the same state as primigravida.^41,42,43^ Physiologic regression presumably plays a role in the phenomenon of parous mothers appearing to have similar birth outcomes as primiparous mothers.

This study showed that a short (<6 months) or long (>36 months) IPI was associated with increased risks of adverse birth outcomes. The findings have substantial implications for public health policy. Many families in China are planning for another child in the context of the shifts in the birth policy.^44^ The findings could inform future public health guidelines on family planning and the interpregnancy period, which is critical given China’s recent adoption of the 3-child policy. As a response to the decline in global fertility rates in recent years, Japan, Germany, Russia, Norway, and other countries have adopted fertility policies to encourage individuals or families to have more children.^45,46,47^ However, pronatalist policies may shorten the interpregnancy interval.^48^ The results of this study imply that other countries may take into account the association between adverse birth outcomes and short IPI when formulating family planning policies.

### Strengths and Limitations

This study has several strengths. First, this cohort study was a large-scale investigation. Second, the matched-sibling method enabled us to incorporate information about first-born siblings into the model to control some unmeasured confounders. If uncontrolled, confounders could lead to the overestimation or underestimation of the risks of adverse birth outcomes and IPI. According to a previous study, mothers with previous adverse birth outcomes are more likely to experience adverse birth outcomes in subsequent pregnancies.^49^ A well-controlled analysis is important in reliably measuring the outcome of IPI. Third, we conducted a more specific analysis to investigate the association between IPI and subcategories of birth outcomes.

This study also has some limitations. First, the study period was restricted from 2014 to 2020, which resulted in a relatively limited amount of data on long IPI (>60 months), making it difficult to explore the association between long IPI and adverse perinatal outcomes. Second, consistent with previous studies,^21,23^ this study included only live, consecutive, single-birth data. Thus, these results might not apply to women who had experienced a miscarriage or stillbirth between deliveries. Given the paucity of guidance on optimal IPI after a stillbirth, further studies on IPI that consider stillbirth are needed. Third, we did not analyze spontaneous PTB and indicated PTB separately because the data were unavailable. Fourth, primary medical institutions report birth surveillance data with varying degrees of lag time. Fifth, there are inherent limitations to retrospective cohort studies because data collection is not controlled by the researchers; this study may not overcome these limitations. These factors may potentially alter the study results.

## Conclusions

This large-scale population-based cohort study conducted in Guangdong Province, China, found that a short IPI of less than 6 months was associated with greater odds of PTB, LBW, and SGA. The study observed that optimal IPI ranged from 18 to 23 months, indicating a shorter optimal IPI than a previous recommendation (24 months). These findings can inform family planning policies and guide individuals and families planning on conceiving another child in China. Further high-quality studies are necessary to explore the association between IPI and adverse birth outcomes in different populations and to examine the underlying mechanisms.

## Notes

Supplement. **eAppendix.** Methods **eTable 1.** Risks of Adverse Birth Outcomes in Unmatched Cohort Analysis, by Detailed Category and Interpregnancy Interval, Guangdong, China, 2014-2020 **eTable 2.** Risks of Adverse Birth Outcomes in Matched-Sibling Analysis, by Detailed Category and Interpregnancy Interval, Guangdong, China, 2014-2020 **eTable 3.** Risks of Adverse Birth Outcomes in Unmatched Cohort Analysis Restricted to Discordant Sibling Pairs, by Interpregnancy Interval, Guangdong, China, 2014-2020 **eTable 4.** Risks of Adverse Birth Outcomes in Unmatched Cohort Analysis by Interpregnancy Interval, Guangdong, China, 2014-2020 **eTable 5.** Risks of Adverse Birth Outcomes in Matched-Sibling Analysis by Interpregnancy Interval, Guangdong, China, 2014-2020 **eFigure 1.** Risks of Adverse Birth Outcomes in Matched-Sibling Analysis and Restricted to Each Subgroup of Delivery Mode of Index Birth, by Interpregnancy Interval, Guangdong, China, 2014-2020 **eFigure 2.** Risks of Adverse Birth Outcomes in Matched-Sibling Analysis and Restricted to Each Subgroup of Maternal Age of Index Birth, by Interpregnancy Interval, Guangdong, China, 2014-2020
