# Supplementary material for: Association of Interpregnancy Interval With Adverse Birth Outcomes
Source: JAMA Netw Open. 2022 Jun 13;5(6):e2216658. doi: 10.1001/jamanetworkopen.2022.16658 (PMC9194661; doi:10.1001/jamanetworkopen.2022.16658)
Supplement: Supplement. — eAppendix. Methods eTable 1. Risks of Adverse Birth Outcomes in Unmatched Cohort Analysis, by Detailed Category and Interpregnancy Interval, Guangdong, China, 2014-2020 eTable 2. Risks of Adverse Birth Outcomes in Matched-Sibling Analysis, by Detailed Category and Interpregnancy Interval, Guangdong, China, 2014-2020 eTable 3. Risks of Adverse Birth Outcomes in Unmatched Cohort Analysis Restricted to Discordant Sibling Pairs, by Interpregnancy Interval, Guangdong, China, 2014-2020 eTable 4. Risks of Adverse Birth Outcomes in Unmatched Cohort Analysis by Interpregnancy Interval, Guangdong, China, 2014-2020 eTable 5. Risks of Adverse Birth Outcomes in Matched-Sibling Analysis by Interpregnancy Interval, Guangdong, China, 2014-2020 eFigure 1. Risks of Adverse Birth Outcomes in Matched-Sibling Analysis and Restricted to Each Subgroup of Delivery Mode of Index Birth, by Interpregnancy Interval, Guangdong, China, 2014-2020 eFigure 2. Risks of Adverse Birth Outcomes in Matched-Sibling Analysis and Restricted to Each Subgroup of Maternal Age of Index Birth, by Interpregnancy Interval, Guangdong, China, 2014-2020 [file jamanetwopen-e2216658-s001.pdf]

Supplemental Online Content

Xu T, Miao H, Chen Y, Luo L, Guo P, Zhu Y. Association of interpregnancy interval with adverse birth outcomes. *JAMA Netw Open*. 2022;5(6):e2216658. doi:10.1001/jamanetworkopen.2022.16658

eAppendix. Methods

eTable 1. Risks of Adverse Birth Outcomes in Unmatched Cohort Analysis, by Detailed Category and Interpregnancy Interval, Guangdong, China, 2014-2020

eTable 2. Risks of Adverse Birth Outcomes in Matched-Sibling Analysis, by Detailed Category and Interpregnancy Interval, Guangdong, China, 2014-2020

eTable 3. Risks of Adverse Birth Outcomes in Unmatched Cohort Analysis Restricted to Discordant Sibling Pairs, by Interpregnancy Interval, Guangdong, China, 2014-2020

eTable 4. Risks of Adverse Birth Outcomes in Unmatched Cohort Analysis by Interpregnancy Interval, Guangdong, China, 2014-2020

eTable 5. Risks of Adverse Birth Outcomes in Matched-Sibling Analysis by Interpregnancy Interval, Guangdong, China, 2014-2020

eFigure 1. Risks of Adverse Birth Outcomes in Matched-Sibling Analysis and Restricted to Each Subgroup of Delivery Mode of Index Birth, by Interpregnancy Interval, Guangdong, China, 2014-2020

eFigure 2. Risks of Adverse Birth Outcomes in Matched-Sibling Analysis and Restricted to Each Subgroup of Maternal Age of Index Birth, by Interpregnancy Interval, Guangdong, China, 2014-2020

This supplemental material has been provided by the authors to give readers additional information about their work.

© 2022 Xu T et al. *JAMA Network Open.*

eAppendix. Methods

The logistic regression model is as follows:

$$\log it(p_i) = X_i^T \beta + \sum_{j=1}^{6} \theta_j IPI_i (IPI_i \in c_j)$$

where p_i is the probability of an adverse birth outcome of interest for the second child from pair i. X_i represents covariates for pair i, such as intercept, maternal age, ethnicity, socioeconomic status, mode of delivery, neonate's sex and birth season. β represents regression coefficients for covariates. IPI is a categorical variable consisting of 7 categories ($c_1 = [0,6)$, $c_2 = [6,11]$, $c_3 = [12,17]$, $c_4 = [24,29]$, $c_5 = [30,35]$, $c_6 = [36,\infty)$ months), with $[18,23]$ months as the reference category. θ_j is a parameter described the association between $IPI_i \in c_j$ and adverse birth outcome of interest for the second-born infant from pair i.

The conditional logistic regression model was the following form:

$$\log it(p_{ij}) = \alpha_i + X_{ij}^T \beta + \sum_{k=1}^{6} \theta_k IPI_i * I + \lambda I$$

where p_{ij} is the probability of adverse birth outcome of interest for birth j from pair i. α_i represents intercept specific to sibling pair i. X_{ij} represents covariates related to birth j from pair i such as maternal age, mode of delivery, neonate's sex and season of birth. β is a regression coefficients vector for covariates. I represents an indicator function that is equal to 0 for the first birth and 1 for the second birth. λ is a regression coefficient explaining the relationship of birth order to a specific adverse outcome of interest.

© 2022 Xu T et al. *JAMA Network Open.*

eTable 1. Risks of Adverse Birth Outcomes in Unmatched Cohort Analysis, by Detailed Category and Interpregnancy Interval, Guangdong, China, 2014-2020

Birth Outcome and Interpregnancy Interval, months	No. of Sibling Pairs	Second Born with Outcome	Crude OR[a]	Adjusted OR[b]
		No. (%)	OR (95% CI)	OR (95% CI)
Moderate preterm birth (32-36 weeks)				
<6	48956	2606 (5.32)	1.59 (1.51-1.67)	1.81 (1.72-1.91)
6-11	173665	6791 (3.91)	1.15 (1.11-1.20)	1.28 (1.23-1.34)
12-17	180429	6373 (3.53)	1.04 (1.00-1.08)	1.10 (1.06-1.15)
18-23	122362	4174 (3.41)	1 [Referent]	1 [Referent]
24-29	86474	3053 (3.53)	1.04 (0.99-1.09)	0.99 (0.94-1.04)
30-35	55101	1956 (3.55)	1.04 (0.99-1.10)	0.97 (0.92-1.02)
≥36	54690	2221 (4.06)	1.20 (1.14-1.26)	1.08 (1.02-1.14)
Very preterm birth (28-31 weeks)				
<6	46862	512 (1.09)	2.71 (2.40-3.08)	3.33 (2.93-3.79)
6-11	167846	972 (0.58)	1.43 (1.28-1.60)	1.68 (1.51-1.88)
12-17	174758	702 (0.40)	0.99 (0.88-1.11)	1.08 (0.96-1.22)
18-23	118669	481 (0.41)	1 [Referent]	1 [Referent]
24-29	83767	346 (0.41)	1.02 (0.89-1.17)	0.95 (0.83-1.09)
30-35	53393	248 (0.46)	1.15 (0.98-1.34)	1.03 (0.88-1.20)
≥36	52752	283 (0.54)	1.33 (1.14-1.54)	1.14 (0.98-1.32)
Extreme preterm birth (< 28 weeks)				
<6	46367	17 (0.04)	1.89 (1.01-3.53)	2.11 (1.11-3.99)
6-11	166912	38 (0.02)	1.17 (0.70-1.96)	1.26 (0.74-2.12)
12-17	174097	41 (0.02)	1.21 (0.73-2.02)	1.26 (0.76-2.11)
18-23	118211	23 (0.02)	1 [Referent]	1 [Referent]
24-29	83443	22 (0.03)	1.36 (0.76-2.43)	1.30 (0.72-2.33)
30-35	53160	15 (0.03)	1.45 (0.76-2.78)	1.33 (0.69-2.57)
≥36	52484	15 (0.03)	1.47 (0.77-2.82)	1.31 (0.68-2.54)
Very low birth weight (< 1500 g)				
<6	46752	180 (0.39)	2.59 (2.11-3.19)	3.37 (2.73-4.18)
6-11	167683	354 (0.21)	1.42 (1.18-1.70)	1.74 (1.44-2.09)
12-17	174951	272 (0.16)	1.04 (0.86-1.26)	1.16 (0.96-1.41)
18-23	118853	177 (0.15)	1 [Referent]	1 [Referent]
24-29	84108	147 (0.17)	1.17 (0.94-1.46)	1.08 (0.87-1.35)
30-35	53594	124 (0.23)	1.56 (1.24-1.96)	1.37 (1.08-1.72)
≥36	52937	146 (0.28)	1.85 (1.49-2.31)	1.56 (1.25-1.95)
Extreme low birth weight (< 1000 g)				
<6	46578	6 (0.01)	1.39 (0.51-3.76)	2.13 (0.77-5.85)
6-11	167351	22 (0.01)	1.42 (0.69-2.93)	1.95 (0.93-4.07)
12-17	174697	18 (0.01)	1.11 (0.53-2.35)	1.31 (0.62-2.77)

© 2022 Xu T et al. *JAMA Network Open.*

18-23	118687	11 (0.01)	1 [Referent]	1 [Referent]
24-29	83976	15 (0.02)	1.93 (0.89-4.20)	1.70 (0.78-3.71)
30-35	53484	14 (0.03)	2.83 (1.28-6.22)	2.29 (1.04-5.06)
≥36	52811	20 (0.04)	4.09 (1.96-8.53)	3.06 (1.45-6.45)

Abbreviations: CI, confidence interval; OR, odds ratio.

[a] OR and corresponding 95% CI based on logistic regression.

[b] Adjusted for maternal age, ethnicity, socioeconomic status, mode of delivery, neonate's sex and season of birth.

© 2022 Xu T et al. *JAMA Network Open.*

eTable 2. Risks of Adverse Birth Outcomes in Matched-Sibling Analysis, by Detailed Category and Interpregnancy Interval, Guangdong, China, 2014-2020

Birth Outcome and Interpregnancy Interval, months	No. of Sibling Pairs	No. of Discordant Pairs in Analysis[c]	Crude IOR[a]	Adjusted IOR[b]
			IOR (95% CI)	IOR (95% CI)
Moderate preterm birth (32-36 weeks)				
<6	48956	3818	1.38 (1.28-1.49)	1.35 (1.25-1.47)
6-11	173665	10667	1.25 (1.17-1.32)	1.22 (1.15-1.30)
12-17	180429	10671	1.09 (1.03-1.16)	1.08 (1.02-1.15)
18-23	122362	7198	1 [Referent]	1[Referent]
24-29	86474	5277	0.99 (0.92-1.06)	1.00 (0.93-1.07)
30-35	55101	3481	0.95 (0.87-1.03)	0.94 (0.87-1.02)
≥36	54690	3587	1.11 (1.02-1.20)	1.09 (1.01-1.19)
Very preterm birth (28-31 weeks)				
<6	46862	600	1.69 (1.35-2.12)	1.68 (1.33-2.12)
6-11	167846	1149	1.89 (1.56-2.29)	1.87 (1.53-2.28)
12-17	174758	953	1.37 (1.12-1.67)	1.36 (1.11-1.66)
18-23	118669	710	1 [Referent]	1 [Referent]
24-29	83767	515	0.99 (0.79-1.25)	1.02 (0.81-1.28)
30-35	53393	358	0.98 (0.76-1.27)	0.98 (0.76-1.27)
≥36	52752	389	1.14 (0.89-1.46)	1.11 (0.86-1.44)
Extreme preterm birth (< 28 weeks)				
<6	46367	32	1.13 (0.42-3.04)	1.19 (0.41-3.43)
6-11	166912	56	1.48 (0.62-3.55)	1.98 (0.77-5.10)
12-17	174097	53	1.81 (0.75-4.40)	2.09 (0.79-5.52)
18-23	118211	32	1 [Referent]	1 [Referent]
24-29	83443	31	2.70 (0.97-7.54)	2.79 (0.92-8.44)
30-35	53160	17	0.90 (0.27-2.96)	1.07 (0.29-3.92)
≥36	52484	16	3.86 (1.02-14.58)	5.00 (1.08-23.24)
Very low birth weight (< 1500 g)				
<6	46752	224	1.40 (0.98-2.00)	1.35 (0.94-1.95)
6-11	167683	449	1.82 (1.34-2.47)	1.80 (1.32-2.47)
12-17	174951	370	1.39 (1.02-1.90)	1.38 (1.00-1.89)
18-23	118853	279	1 [Referent]	1 [Referent]
24-29	84108	236	1.05 (0.74-1.48)	1.05 (0.74-1.49)
30-35	53594	161	1.12 (0.76-1.66)	1.19 (0.81-1.77)
≥36	52937	185	1.90 (1.29-2.78)	1.98 (1.33-2.95)
Extreme low birth weight (< 1000 g)				
<6	46578	17	0.40 (0.10-1.61)	0.37 (0.09-1.55)
6-11	167351	36	1.16 (0.41-3.33)	1.23 (0.41-3.74)
12-17	174697	32	0.89 (0.30-2.63)	1.00 (0.32-3.11)

© 2022 Xu T et al. *JAMA Network Open.*

18-23	118687	23	1 [Referent]	1 [Referent]
24-29	83976	22	1.88 (0.57-6.14)	2.32 (0.67-8.07)
30-35	53484	16	2.17 (0.59-7.99)	2.18 (0.57-8.37)
≥36	52811	19	3.64 (0.98-13.52)	5.44 (1.23-23.93)

Abbreviations: CI, confidence interval; IOR, interaction odds ratio.

[a] Odds of second-born child being born with outcome under analysis compared with odds of first being born with same outcome.

[b] Adjusted for maternal age, mode of delivery, neonate's sex and season of birth.

[c] Sibling pairs with exactly one birth resulting in an adverse outcome under analysis.

© 2022 Xu T et al. *JAMA Network Open.*

eTable 3. Risks of Adverse Birth Outcomes in Unmatched Cohort Analysis Restricted to Discordant Sibling Pairs, by Interpregnancy Interval, Guangdong, China, 2014-2020

Birth Outcome and Interpregnancy Interval, months	No. of Discordant Sibling Pairs[c]	Second born with outcome	Crude OR[a]	Adjusted OR[b]
		No. (%)	OR (95% CI)	OR (95% CI)
Preterm birth (< 37 weeks)				
<6	4450	2590 (58.20)	1.43 (1.33-1.54)	1.58 (1.47-1.71)
6-11	11872	6627 (55.82)	1.30 (1.23-1.37)	1.39 (1.31-1.47)
12-17	11677	6067 (51.96)	1.11 (1.05-1.18)	1.15 (1.08-1.22)
18-23	7940	3919 (49.36)	1 [Referent]	1 [Referent]
24-29	5823	2869 (49.27)	1.00 (0.93-1.07)	0.97 (0.90-1.04)
30-35	3856	1854 (48.08)	0.95 (0.88-1.03)	0.92 (0.85-1.00)
≥36	3992	2081 (52.13)	1.12 (1.04-1.21)	1.06 (0.98-1.14)
Moderate preterm birth (32-36 weeks)				
<6	3818	2171 (56.86)	1.38 (1.28-1.50)	1.53 (1.41-1.65)
6-11	10667	5796 (54.34)	1.25 (1.17-1.32)	1.33 (1.26-1.42)
12-17	10671	5440 (50.98)	1.09 (1.03-1.16)	1.13 (1.06-1.20)
18-23	7198	3515 (48.83)	1 [Referent]	1 [Referent]
24-29	5277	2566 (48.63)	0.99 (0.92-1.07)	0.96 (0.89-1.03)
30-35	3481	1652 (47.46)	0.95 (0.87-1.03)	0.92 (0.84-0.99)
≥36	3587	1843 (51.38)	1.11 (1.02-1.20)	1.04 (0.96-1.13)
Very preterm birth (28-31 weeks)				
<6	600	404 (67.33)	1.69 (1.35-2.12)	1.97 (1.56-2.50)
6-11	1149	801 (69.71)	1.89 (1.56-2.29)	2.09 (1.71-2.56)
12-17	953	596 (62.54)	1.37 (1.12-1.67)	1.45 (1.19-1.77)
18-23	710	390 (54.93)	1 [Referent]	1 [Referent]
24-29	515	282 (54.76)	0.99 (0.79-1.25)	0.98 (0.78-1.24)
30-35	358	195 (54.47)	0.98 (0.76-1.27)	0.95 (0.74-1.23)
≥36	389	226 (58.10)	1.14 (0.89-1.46)	1.10 (0.85-1.42)
Extreme preterm birth (< 28 weeks)				
<6	32	15 (46.88)	1.13 (0.42-3.04)	0.98 (0.33-2.90)
6-11	56	30 (53.57)	1.48 (0.62-3.56)	1.64 (0.64-4.21)
12-17	53	31 (58.49)	1.81 (0.75-4.40)	1.80 (0.69-4.69)
18-23	32	14 (43.75)	1 [Referent]	1 [Referent]
24-29	31	21 (67.74)	2.70 (0.97-7.54)	3.00 (1.00-9.04)
30-35	17	7 (41.18)	0.90 (0.27-2.96)	0.85 (0.24-3.04)
≥36	16	12 (75.00)	3.86 (1.02-14.58)	4.63 (1.10-19.60)
Low birth weight (< 2500 g)				
<6	4780	2229 (46.63)	1.32 (1.23-1.42)	1.42 (1.32-1.53)
6-11	12792	5901 (46.13)	1.29 (1.22-1.37)	1.37 (1.29-1.45)

© 2022 Xu T et al. *JAMA Network Open.*

12-17	12171	5176 (42.53)	1.12 (1.06-1.18)	1.15 (1.09-1.22)
18-23	8339	3323 (39.85)	1 [Referent]	1 [Referent]
24-29	5955	2297 (38.57)	0.95 (0.89-1.02)	0.92 (0.86-0.99)
30-35	3743	1471 (39.30)	0.98 (0.90-1.06)	0.94 (0.86-1.01)
≥36	3969	1737 (43.76)	1.18 (1.09-1.27)	1.12 (1.03-1.21)
Very low birth weight (< 1500 g)				
<6	224	132 (58.93)	1.40 (0.99-2.00)	1.50 (1.04-2.17)
6-11	449	292 (65.03)	1.82 (1.34-2.47)	1.92 (1.40-2.63)
12-17	370	217 (58.65)	1.39 (1.02-1.90)	1.43 (1.04-1.96)
18-23	279	141 (50.54)	1 [Referent]	1 [Referent]
24-29	236	122 (51.69)	1.05 (0.74-1.48)	1.04 (0.74-1.48)
30-35	161	86 (53.42)	1.12 (0.76-1.66)	1.12 (0.76-1.66)
≥36	185	122 (65.95)	1.90 (1.29-2.78)	1.99 (1.34-2.95)
Extreme low birth weight (< 1000 g)				
<6	17	4 (23.53)	0.40 (0.10-1.61)	0.26 (0.05-1.26)
6-11	36	17 (47.22)	1.16 (0.41-3.33)	1.07 (0.32-3.55)
12-17	32	13 (40.63)	0.89 (0.30-2.63)	0.77 (0.22-2.65)
18-23	23	10 (43.48)	1 [Referent]	1 [Referent]
24-29	22	13 (59.09)	1.88 (0.58-6.14)	2.37 (0.64-8.84)
30-35	16	10 (62.50)	2.17 (0.59-7.99)	2.36 (0.55-10.05)
≥36	19	14 (73.68)	3.64 (0.98-13.52)	4.87 (1.09-21.79)
Small for gestational age				
<6	11143	4139 (37.14)	1.17 (1.11-1.22)	1.17 (1.12-1.23)
6-11	35835	13524 (37.74)	1.20 (1.16-1.24)	1.20 (1.16-1.25)
12-17	34971	12240 (35.00)	1.06 (1.03-1.10)	1.06 (1.03-1.10)
18-23	22455	7552 (33.63)	1 [Referent]	1 [Referent]
24-29	15333	4999 (32.60)	0.96 (0.91-1.00)	0.95 (0.91-0.99)
30-35	9448	3122 (33.04)	0.97 (0.93-1.03)	0.97 (0.92-1.02)
≥36	9427	2957 (31.37)	0.90 (0.86-0.95)	0.90 (0.86-0.95)

Abbreviations: CI, confidence interval; OR, odds ratio.

[a] OR and corresponding 95% CI based on logistic regression.

[b] Adjusted for maternal age, ethnicity, socioeconomic status, mode of delivery, neonate's sex and season of birth.

[c] Sibling pairs with exactly one birth resulting in an adverse outcome under analysis.

© 2022 Xu T et al. *JAMA Network Open.*

eTable 4. Risks of Adverse Birth Outcomes in Unmatched Cohort Analysis by Interpregnancy Interval, Guangdong, China, 2014-2020

Birth Outcome and Interpregnancy Interval, months	No. of Sibling Pairs	Second Born with Outcome	Crude OR[a]	Adjusted OR[b]
		No. (%)	OR (95% CI)	OR (95% CI)
Preterm birth				
<6	49485	3135 (6.34)	1.71 (1.63-1.79)	1.73 (1.65-1.81)
6-11	174675	7801 (4.47)	1.18 (1.14-1.23)	1.19 (1.15-1.24)
12-17	181172	7116 (3.93)	1.03 (1.00-1.07)	1.04 (1.00-1.08)
18-23	122866	4678 (3.81)	1 [Referent]	1 [Referent]
24-29	86842	3421 (3.94)	1.04 (0.99-1.08)	1.03 (0.98-1.07)
30-35	55364	2219 (4.01)	1.06 (1.00-1.11)	1.03 (0.98-1.09)
≥36	54988	2519 (4.58)	1.21 (1.15-1.28)	1.16 (1.10-1.22)
Low birth weight				
<6	49485	2913 (5.89)	1.77 (1.69-1.86)	1.70 (1.61-1.78)
6-11	174675	7346 (4.21)	1.24 (1.20-1.29)	1.21 (1.16-1.26)
12-17	181172	6493 (3.58)	1.05 (1.01-1.10)	1.04 (1.00-1.08)
18-23	122866	4190 (3.41)	1 [Referent]	1 [Referent]
24-29	86842	2881 (3.32)	0.97 (0.93-1.02)	0.98 (0.93-1.03)
30-35	55364	1894 (3.42)	1.00 (0.95-1.06)	1.01 (0.96-1.07)
≥36	54988	2197 (4)	1.18 (1.12-1.24)	1.19 (1.13-1.26)
Small for gestational age				
<6	49444	7472 (15.11)	1.49 (1.44-1.53)	1.36 (1.32-1.40)
6-11	174611	23522 (13.47)	1.30 (1.27-1.33)	1.22 (1.19-1.25)
12-17	181121	21338 (11.78)	1.11 (1.09-1.14)	1.08 (1.05-1.10)
18-23	122844	13145 (10.7)	1 [Referent]	1 [Referent]
24-29	86829	8770 (10.1)	0.94 (0.91-0.97)	0.97 (0.94-0.99)
30-35	55350	5390 (9.74)	0.90 (0.87-0.93)	0.95 (0.92-0.98)
≥36	54983	5175 (9.41)	0.87 (0.84-0.90)	0.95 (0.92-0.98)

Abbreviations: CI, confidence interval; OR, odds ratio.

[a] OR and corresponding 95% CI based on logistic regression.

[b] Adjusted for maternal age, ethnicity, socioeconomic status, neonate’s sex and season of birth.

© 2022 Xu T et al. *JAMA Network Open.*

eTable 5. Risks of Adverse Birth Outcomes in Matched-Sibling Analysis by Interpregnancy Interval, Guangdong, China, 2014-2020

Birth Outcome and Interpregnancy Interval, months	No. of Sibling Pairs	No. of Discordant Pairs in Analysis[c]	Crude IOR[a]	Adjusted IOR[b]
			IOR (95% CI)	IOR (95% CI)
Preterm birth				
<6	49485	4450	1.43 (1.33-1.54)	1.42 (1.31-1.53)
6-11	174675	11872	1.30 (1.22-1.37)	1.29 (1.22-1.36)
12-17	181172	11677	1.11 (1.05-1.17)	1.11 (1.05-1.18)
18-23	122866	7940	1 [Referent]	1 [Referent]
24-29	86842	5823	1.00 (0.93-1.07)	1.00 (0.93-1.07)
30-35	55364	3856	0.95 (0.88-1.03)	0.95 (0.87-1.02)
≥36	54988	3992	1.12 (1.04-1.21)	1.12 (1.03-1.21)
Low birth weight				
<6	49485	4780	1.32 (1.23-1.42)	1.32 (1.23-1.42)
6-11	174675	12792	1.29 (1.22-1.37)	1.30 (1.22-1.37)
12-17	181172	12171	1.12 (1.06-1.18)	1.12 (1.06-1.18)
18-23	122866	8339	1 [Referent]	1 [Referent]
24-29	86842	5955	0.95 (0.89-1.01)	0.94 (0.88-1.01)
30-35	55364	3743	0.98 (0.90-1.06)	0.98 (0.91-1.06)
≥36	54988	3969	1.17 (1.09-1.27)	1.17 (1.09-1.27)
Small for gestational age				
<6	49444	11143	1.17 (1.11-1.22)	1.16 (1.11-1.22)
6-11	174611	35835	1.20 (1.16-1.24)	1.19 (1.15-1.23)
12-17	181121	34971	1.06 (1.03-1.10)	1.05 (1.02-1.09)
18-23	122844	22455	1 [Referent]	1 [Referent]
24-29	86829	15333	0.95 (0.91-1.00)	0.95 (0.91-0.99)
30-35	55350	9448	0.97 (0.93-1.02)	0.98 (0.93-1.03)
≥36	54983	9427	0.90 (0.86-0.95)	0.90 (0.85-0.95)

Abbreviations: CI, confidence interval; IOR, interaction odds ratio.

[a] Odds of second-born child being born with outcome under analysis compared with odds of first being born with same outcome.

[b] Adjusted for maternal age, neonate's sex and season of birth.

[c] Sibling pairs with exactly one birth resulting in an adverse outcome under analysis.

© 2022 Xu T et al. *JAMA Network Open.*

eFigure 1. Risks of Adverse Birth Outcomes in Matched-Sibling Analysis and Restricted to Each Subgroup of Delivery Mode of Index Birth, by Interpregnancy Interval, Guangdong, China, 2014-2020

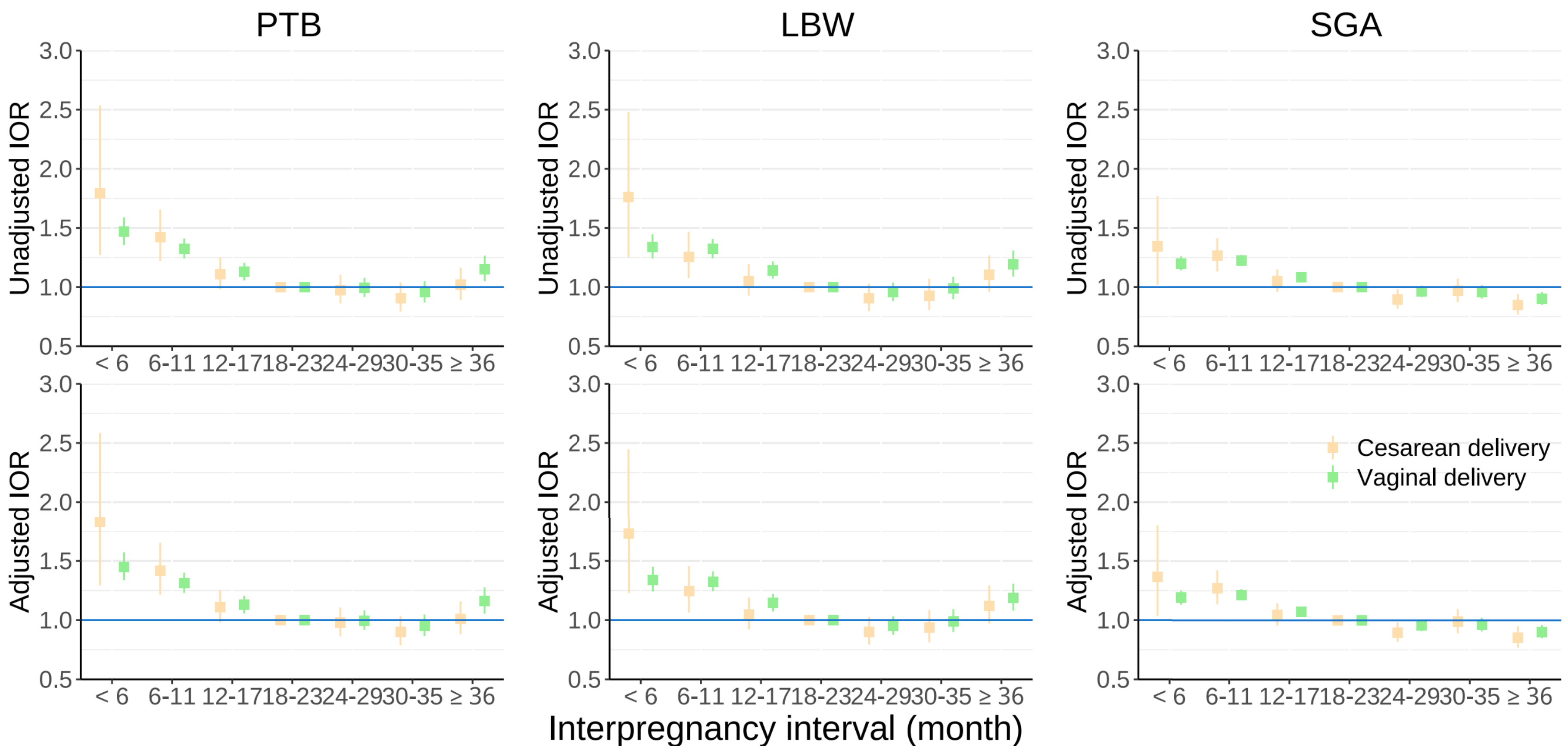

© 2022 Xu T et al. *JAMA Network Open.*

eFigure 2. Risks of Adverse Birth Outcomes in Matched-Sibling Analysis and Restricted to Each Subgroup of Maternal Age of Index Birth, by Interpregnancy Interval, Guangdong, China, 2014-2020

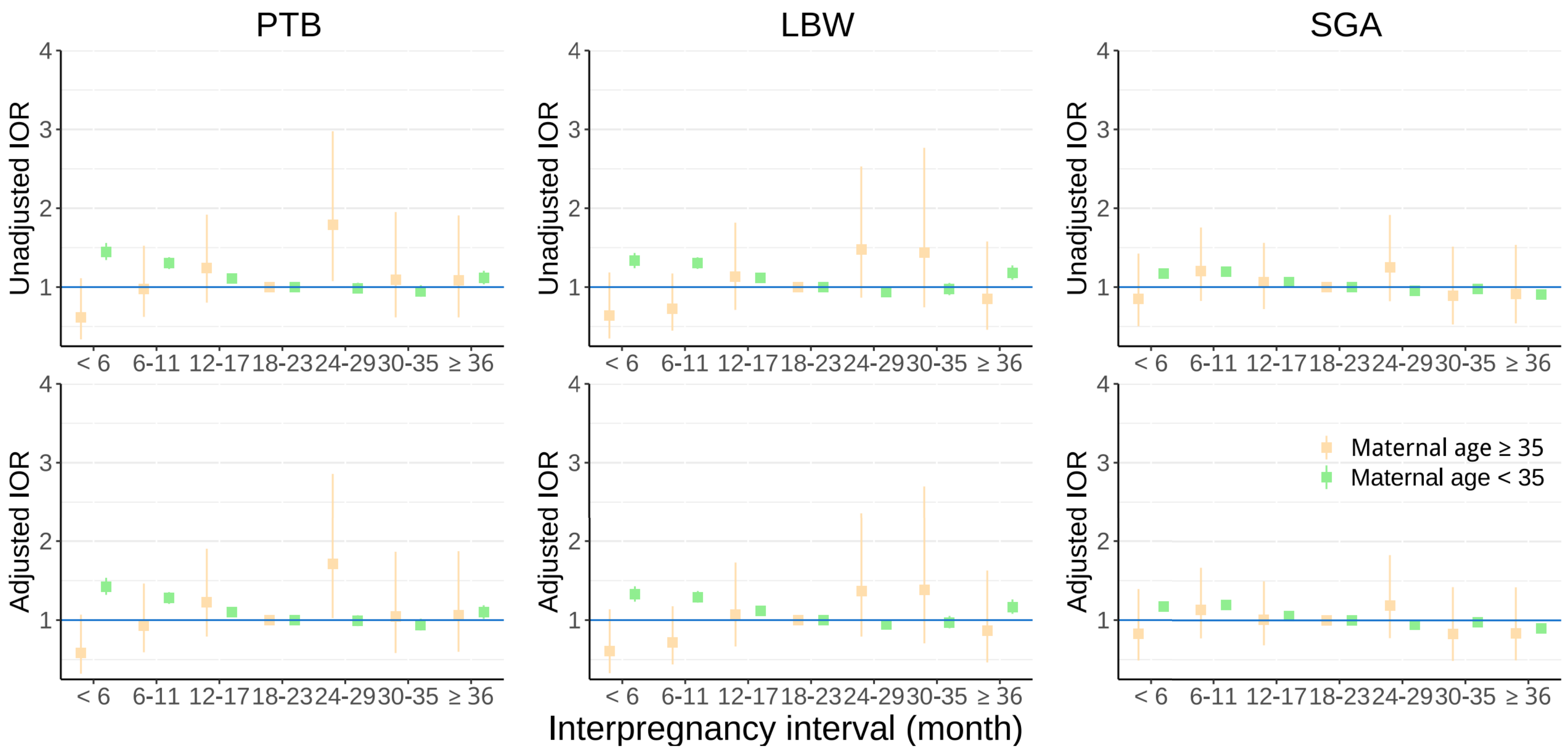

© 2022 Xu T et al. *JAMA Network Open.*
